# Association between access to health-promoting facilities and participation in cardiovascular disease (CVD) risk screening among populations with low socioeconomic status (SES) in Singapore

**DOI:** 10.1017/S1463423619000318

**Published:** 2019-07-01

**Authors:** Ka Keat Lim, Charmaine Lim, Yu Heng Kwan, Sui Yung Chan, Warren Fong, Lian Leng Low, Hung Yong Tay, Truls Østbye, Chuen Seng Tan

**Affiliations:** 1 Programme in Health Services & Systems Research, Duke-NUS Medical School, National University of Singapore, Singapore, Republic of Singapore; 2 Department of Pharmacy, Faculty of Science, National University of Singapore, Singapore, Republic of Singapore; 3 Department of Rheumatology and Immunology, Singapore General Hospital, Singapore, Republic of Singapore; 4 Department of Medicine, Yong Loo Lin School of Medicine, National University of Singapore, Singapore, Republic of Singapore; 5 Duke-NUS Medical School, Singapore, Republic of Singapore; 6 Department of Family Medicine & Continuing Care, Singapore General Hospital, Singapore, Republic of Singapore; 7 Singapore Heart Foundation, Singapore, Republic of Singapore; 8 Saw Swee Hock School of Public Health, National University of Singapore, Singapore, Republic of Singapore

**Keywords:** cardiovascular disease, medical screening, prevention, socioeconomic status, screening

## Abstract

**Background:**

Low socioeconomic status (SES) is a barrier for cardiovascular disease (CVD) risk screening and a determinant of poor CVD outcomes. This study examined the associations between access to health-promoting facilities and participation in a CVD risk screening program among populations with low SES residing in public rental flats in Singapore.

**Methods:**

Data from Health Mapping Exercises conducted from 2013 to 2015 were obtained, and screening participation rates of 66 blocks were calculated. Negative binomial regression was used to test for associations between distances to four nearest facilities (i.e., subsidized private clinics, healthy eateries, public polyclinics, and parks) and block participation rate in CVD screening. We also investigated potential heterogeneity in the association across regions with an interaction term between distance to each facility and region.

**Results:**

The analysis consisted of 2069 participants. The associations were only evident in the North/North-East region for subsidized private clinic and park. Specifically, increasing distance to the nearest subsidized private clinic and park was significantly associated with lower [incidence rate ratio (IRR) = 0.88, 95% confidence interval (CI): 0.80–0.98] and higher (IRR = 1.93, 95%CI: 1.15–3.25) screening participation rates respectively.

**Conclusions:**

Our findings could potentially inform the planning of future door-to-door screenings in urban settings for optimal prioritization of resources. To increase participation rates in low SES populations, accessibility to subsidized private clinics and parks in a high population density region should be considered.

## Introduction

The inverse relationship between socioeconomic status (SES) and cardiovascular disease (CVD) has long been established (Backholer *et al.*, 2017) – populations with low SES not only have poorer knowledge on CVD risk factors (Tsuji *et al.*, 2018), but also have poorer control of the risk factors (Leng *et al.*, 2015), resulting in higher CVD incidence and mortality rates than populations with high SES (Woodward *et al.*, 2015).

Pharmacological interventions (Chou *et al.*, 2016) and lifestyle changes (Sisti *et al.*, 2018) could control CVD risk factors and reduce mortality. Thus, early screening for CVD risk factors and risk modification are crucial in reducing complications and deaths. Various modes of CVD risk screening are available, ranging from door-to-door (Wee *et al.*, 2013; US Office of Disease Prevention and Health Promotion (ODPHP), 2019), healthcare facility-based (Baker *et al.*, 2018), workplace-based (Abbas *et al.*, 2015), to outreach program (Zhang *et al.*, 2017). However, a low SES has also been shown to be a barrier to CVD risk screening. In addition, studies examined mostly individual-level factors associated with CVD risk screening participation, for example, age, marital status, education level, and attitudes (Groenenberg *et al.*, 2015). While individual-level determinants may advise targeted interventions for potential non-screening participants, awareness of the environmental determinants could help policy makers and healthcare providers to prioritize areas during the planning phase of future screening program in socio-economically disadvantaged neighborhoods.

Associations between one’s physical environment and health behaviors are well reported in literature. For instance, poor access to healthy food and recreational facilities were shown to be associated with poor dietary habits (Caspi *et al.*, 2012) and low levels of physical activity (Perez *et al.*, 2018; Zhang *et al.*, 2018) respectively. Nevertheless, to the best of our knowledge, no study has examined the association between physical environment and CVD risk screening participation among populations with low SES.

This study bridged the evidence gap by investigating the associations between distances from public rental flats to the nearest of the four health-promoting facilities (i.e., subsidized private clinic, polyclinic, healthy eatery, and park) and participation rate in a door-to-door CVD risk screening, using public rental flat in Singapore as the unit of analysis. We further tested potential heterogeneity in the association by considering an interaction between the distance from the nearest health-promoting facility and region. Singapore is a city state in South-East Asia, comprising three major ethnic groups (Chinese, Malays, and Indian). Public rental flats (Housing & Development Board Singapore, 2013; Housing & Development Board Singapore, 2018), embedded within the same precinct as per owner-occupied public housing, are provided for those who do not own asset and with household income < SGD1,500 (approximately USD1,100, the lowest decile income group versus average SGD9,023 or USD6,600 (Department of Statistics Singapore, 2018). The findings could be important in planning future door-to-door screenings among lower-income populations in urban settings. We hypothesized that poor access to health-promoting facilities in the neighborhood is associated with poor health screening behavior. Therefore, with increasing distance to the nearest health-promoting facilities, we expect a decrease in block screening participation rate.

## Methods

### Study design

We performed secondary data analyses based on the Health Mapping Exercises (HME) database in Singapore Heart Foundation. HME (Singapore Heart Foundation, 2018) was an annual, free, door-to-door, CVD risk screening program for residents living in one- or two-room apartments in Housing Development Board (HDB) public rental flats (hereafter referred to as ‘blocks’). In HME, trained volunteers in SHF screened the residents for dietary and lifestyle risk factors such as smoking, alcohol consumption, fruits and vegetable consumption, blood pressure, and body mass index. We included all blocks screened by HME in the present study as there was no sample size calculation performed before data collection. We obtained ethics approval from the National University of Singapore Institutional Review Board (IRB Number: B-16-085E) to use the data for this study.

### Data sources

We used data on door-to-door screening uptake of HME between 2013 and 2015. Data at first screening were used, as the majority of participants (94.3%) had a single encounter.

Location of each block was acquired from HDB Map Services (Housing & Development Board Singapore, 2019). Location of subsidized private clinics, healthy eateries, and parks in Singapore were obtained from OneMap 2016^©^ (Singapore Land Authority, 2016), an integrated map system maintained by multiple government agencies. Location of polyclinics and HDB blocks were identified from official addresses on websites. We estimated the distances from each block to the nearest health-promoting facilities based on these locations.

Polyclinics and subsidized private clinics, which were two out of the four health-promoting facilities considered, are providers of primary care services in Singapore (Khoo *et al.*, 2014). While majority (80%) of primary care visits occur in the private clinics, the polyclinics received higher proportions (40%) of chronic diseases (Ministry of Health Singapore, 2014). Subsidized private clinics refer to private clinics participating in the Community Health Assist Scheme under the Singapore’s Ministry of Health (MOH) (Ministry of Health Singapore, 2018), which provides means-tested subsidies for medical care, dental care, and health screening for elderly Singaporean and citizens from low- to middle-income households.

Healthy eateries in this study refer to food and beverages businesses participating in the Healthier Dining Program by the Singapore Health Promotion Board (HPB) (Health Promotion Board, 2019). Under the certification program, healthy eateries provide healthy food choices, such as low sugar beverages, low calorie meals, and wholegrain options in return for subsidy from the HPB. Meanwhile, parks refer to public spaces with greeneries for recreation in the community.

### Data processing

The HME had reached out to 66 blocks and screened 2619 individuals over the three-year period. Individuals with missing age (self-reported or calculated age from date of birth) or with a difference of greater than or equal to two years between calculated and self-reported ages were excluded from the analyses. Hence, our analyses comprised of 2069 HME participants. To account for demographic factors in the variability of block screening participant rate, participants were aggregated by block to obtain the number of participants stratified by: age (<40 or ≥40 years old), ethnicity (Chinese or non-Chinese), and gender (male or female). We used 40 years old as the age cut-off as Singapore’s MOH recommends CVD risk screening for adults ≥40 years old (Ministry of Health Singapore, 2011). We used QGIS® to estimate distance from the blocks to the nearest facilities as proxy of access, where kilometers (km) were used for parks and polyclinics, and meters (m) were used for subsidized private clinics and healthy eateries. We described the distances to park and polyclinics in kilometers (km) as these facilities were located approximately 1 km away from the public rental flats. Similarly, we described distances to subsidized private clinics and healthy eateries in 100 m, as all these facilities were located within 1 km from the public rental flats. Using km for park and polyclinics and 100 m for subsidized private clinics and healthy eateries also ensured that the IRR could be reported in a reasonable range. The blocks were located across five planning regions in Singapore (North, North-East, East, Central, and West regions, as demarcated by the Urban Redevelopment Authority), with only one block each in the North and West regions. To facilitate analyses, we combined North region with North-East region and West region with Central region based on their proximities.

### Data analysis

The outcome of interest was block screening participation and the exposures of interest were distances to the nearest polyclinics, subsidized private clinics, healthy eateries, and parks. Block screening participation rate (per 100 apartment-years) was calculated by dividing the number of participants in a block (overall or specific demographic characteristics) by three times the total number of one- or two-room apartments in a block, multiplied by 100. We first performed a descriptive analysis on the block characteristics and reported their medians, interquartile ranges (IQR), and minimum and maximum values.

Due to the presence of over-dispersion in the data, we used negative binomial regressions (Lee *et al.*, 2012) to model the block screening participation rate where the total number of participants was the dependent variable with an offset term corresponding to the natural log of the total number of one- or two-room apartments in a block multiplied by three years. We began with a bivariate analysis to assess the association between distance to the nearest facility and block screening participation rate where each of the four health-promoting facilities was analyzed separately (Model 1). Subsequently, we performed a multivariate analysis to adjust for age, gender, ethnicity, and region (Model 2). The previous analyses assume that the effect of distance to the nearest facility on participation rate was homogeneous. To assess whether the effect could be different across regions, we repeated the previous multivariate analysis but with the addition of an interaction term between distance to the nearest facility and region (Model 3). To obtain a parsimonious model (Model 4) with the multiple interaction terms considered in the preceding analysis, we performed a backward variable selection on a model with age, gender, ethnicity, region, and distances to the nearest facilities that had significant interaction term in Model 3 and their interaction terms; we removed the interaction terms sequentially until none of the them had a *P*-value exceeding 0.05.

In all regression analyses, we reported the incidence rate ratios (IRRs) with their respective 95% confidence intervals (CI) and *P*-values. A *P*-value of <0.05 was considered statistically significant.

## Results

### Block characteristics

A total of 66 blocks were in our study, with the majority (53.0%) located in the Central/West regions, and the remaining blocks almost equally distributed between the East (24.2%) and North/North-East (22.7%) regions. These blocks housed a median of 216 one- or two-room apartments (see Table 1). The median distances (IQR) to the nearest subsidized private clinic, healthy eatery, park, and polyclinic were 191.7 m (123.8 m), 334.1 m (265.7 m), 0.8 km (0.7 km), and 2.1 km (2.1 km) respectively. All blocks were within 1 km to the nearest subsidized private clinic (minimum–maximum: 47.5–811.8 m) and healthy eatery (77.2–903.0 m), much closer than to the nearest park (0.2–2.7 km) and polyclinic (0.3–16.7 km). There were small differences in median distance to the nearest subsidized private clinic, healthy eatery, and park between the three regions. In contrast, distance to the nearest polyclinics differed vastly between regions, with shortest distance for blocks in the East region, followed by Central/West and North/North-East regions.

**Table 1.** Characteristics of the blocks that are in the study (*n* = 66 blocks)

Block Characteristic	Median (IQR)	(Minimum–Maximum)
Number of apartments in a block		
One- or two-room apartment	216.0 (110.0)	(9–520.0)
Distance to the nearest facility		
Subsidized private clinic (in m)	191.7 (123.8)	(47.5–811.8)
East region	143.6 (43.5)	(73.5–372.9)
Central/West regions	191.7 (119.9)	(49.2–587.6)
North/North-East regions	230.3 (76.7)	(47.5–811.8)
Healthy eatery (in m)	334.1 (265.7)	(77.2–902.9)
East region	406.8 (223.6)	(132.8–776.0)
Central/West regions	334.1 (259.3)	(77.2–902.9)
North/North-East regions	464.2 (529.1)	(191.9–881.6)
Park (in km)	0.8 (0.7)	(0.2–2.7)
East region	1.1 (0.7)	(0.5–2.2)
Central/West regions	0.8 (0.7)	(0.4–2.7)
North/North-East regions	0.7 (0.7)	(0.2–2.0)
Polyclinic (in km)	2.1 (2.1)	(0.3–16.7)
East region	0.9 (0.4)	(0.7–3.3)
Central/West regions	2.1 (2.1)	(0.3–16.7)
North/North-East regions	3.7 (2.7)	(0.4–14.5)
Block screening participation rate*		
Overall	4.6 (2.9)	(0.1–11.1)
Aged ≥40	8.5 (6.6)	(1.6–49.2)
Aged <40	0.7 (0.8)	(0.0–4.9)
Chinese	5.7 (3.2)	(1.1–30.8)
Non-Chinese	4.0 (3.7)	(0.7–20.0)
Male	4.4 (3.4)	(0.6–20.8)
Female	5.1(3.4)	(1.2–30.0)
Regions		
East region	6.2 (4.8)	(1.6–11.1)
Central/West regions	4.5 (2.7)	(0.1–8.2)
North/North-East regions	4.0 (1.7)	(0.8–5.9)

* Block screening participation rate (per 100 apartment-years) was calculated using the participant count in a block (either overall count or number of participants within a specified socio-demographic characteristic) dividing by three times the total number of one- and two-room apartments in a block and then multiplying by 100 (because HME was a three-year door-to-door screening program). For example, a median participation rate value of 4.5 for males can be interpreted as 4.5 male participants per 100 apartment-years.

The median overall screening participation rate was 4.6 per 100 apartment-years. The median screening participation rate among Chinese, non-Chinese, males, and females were 5.7, 4.0, 4.4, and 5.1 per 100 apartment-years respectively and similar to the overall rate. However, those aged ≥40 had a higher screening participation rate (8.5 per 100 apartment-years) than overall and those aged <40 (0.7 per 100 apartment-years). Meanwhile the East region had the highest rate (6.2 per 100 apartment-years), followed by the Central/West (4.5 per 100 apartment-years) and North/North-East (4.0 per 100 apartment-years) regions.

### Negative binomial regression

For the unadjusted analyses (see Model 1 in Table 2), significant association was observed between blocks’ distance to the nearest polyclinic (IRR 0.94, 95%CI 0.89–0.98) and block screening participation rate. All associations were insignificant after adjusting for age, gender, ethnicity, and region (see Model 2 in Table 2).

**Table 2.** Effect of distance to the nearest facility on block screening participation rate

Homogeneous effect	Model 1					Model 2				
	IRR	(95% CI)			*P*-value	IRR	95% CI			*P*-value
Distance to the nearest:										
Subsidized private clinic (in 100 m)	0.91	(0.82	–	1.01)	0.071	1.02	(0.94	–	1.10)	0.652
Park (in km)	0.92	(0.61	–	1.40)	0.705	1.05	(0.79	–	1.39)	0.762
Healthy eatery (in 100 m)	1.03	(0.95	–	1.13)	0.439	1.03	(0.96	–	1.10)	0.415
Polyclinic (in km)	**0.94**	**(0.89**	**–**	**0.98)**	**0.007**	0.97	(0.94	–	1.00)	0.088

Heterogeneous effect	Model 3					Model 4				
	IRR	(95% CI)			*P*-value	IRR	95% CI			*P*-value
Distance to the nearest subsidized private clinic (by every 100 m)										
East region	**1.52**	**(1.12**	**–**	**2.05)**	**0.007**	1.44	(0.56	**–**	3.67)	0.450
Central/West regions	0.97	(0.83	–	1.13)	0.685	0.98	(0.84	**–**	1.16)	0.852
North/North-East regions	1.02	(0.95	–	1.09)	0.619	**0.88**	**(0.80**	**–**	**0.98)**	**0.017**
Distance to the nearest park (by every km)										
East region	1.38	(0.60	–	3.16)	0.444	1.09	(0.24	**–**	4.94)	0.909
Central/West regions	0.85	(0.60	–	1.22)	0.385	0.83	(0.59	**–**	1.18)	0.308
North/North-East regions	**1.42**	**(1.11**	**–**	**1.81)**	**0.006**	**1.93**	**(1.15**	–	**3.25)**	**0.013**
Distance to the nearest healthy eatery (by every 100 m)										
East region	1.03	0.80	–	1.33	0.824	-	-	-	-	-
Central/West regions	1.01	0.95	–	1.08	0.711	-	-	-	-	-
North/North-East regions	1.06	0.95	–	1.18	0.290	-	-	-	-	-
Distance to the nearest polyclinic (by every km)										
East region	0.81	(0.61	–	1.07)	0.132	-	-	-	-	-
Central/West regions	1.02	(0.97	–	1.06)	0.534	-	-	-	-	-
North/North-East regions	**0.92**	**(0.88**	**–**	**0.96)**	**<0.001**	-	-	-	-	-

IRR = Incidence Rate Ratio.

Model 1: Unadjusted analysis for each facility.

Model 2: Adjusted for age, gender, and ethnicity, with homogeneous effect across regions for each facility.

Model 3: Adjusted for age, gender, and ethnicity with heterogeneous effect across regions for each facility.

Model 4: Adjusted for age, ethnicity, gender, and distance to the nearest polyclinic with heterogeneous effect across regions for private clinic and park.

To assess variability in the association between distance to nearest health-promoting facilities and health screening behavior in different regions, an interaction term between distance and region was included into the multivariate models. The interaction terms of region with distance to the nearest subsidized private clinics, parks, and polyclinics respectively were significant. In particular, distance to the nearest subsidized private clinics in the East region (IRR = 1.52, 95%CI: 1.12–2.05) and parks in the North/North-East regions (IRR = 1.42, 95%CI: 1.11–1.81) were significantly associated with block screening participation rate (see Model 3 in Table 2). Distance to the nearest polyclinic in North/North-East regions (IRR = 0.92, 95%CI: 0.88–0.96) was also significant, albeit in the opposite direction. The parsimonious model included interaction terms for subsidized private clinic and park (see Model 4 in Table 2). Distance to nearest subsidized private clinics (IRR = 0.88, 95%CI: 0.80–0.98) and park (IRR = 1.93, 95%CI: 1.15–3.25) in North/North-East regions were associated with poorer and better block screening participation rate respectively. In the parsimonious model, distance to nearest polyclinic was not associated with block screening participate rate (IRR = 0.98, 95%CI: 0.94−1.01).

## Discussion

To our best knowledge, this is the first study examining the associations between access to health-promoting facilities and participation in door-to-door CVD risk screening in low SES population. The main strength of our study was the integration of data from different sources coupled with robust statistical analyses that accounted for potential confounding and over-dispersion, and considered a parsimonious model where distances to multiple facilities were allowed to be simultaneously in the same model so as to account for potential variation from other facilitates when assessing each facility.

We found that the associations between access to facilities and block screening participation rate varied according to the type of health-promoting facility and region. Increasing distance to the nearest park (every 1 km increase) and to the nearest subsidized private clinic (every 100 m increase) was significantly associated with a 93% increase and 12% decrease in block screening participation rates respectively. Interestingly, these were only observed in the North/North-East regions where all blocks except one were in the North-East, and this region has the highest population density (Department of Statistics Singapore, 2016). Under resource constraint, future screening and health promotion initiatives could target residents with low SES in the region living far from subsidized private clinics but close to a park in high population density region. Future work should identify potential factors that could explain the variation in participation rate across regions observed.

Our finding on subsidized private clinic is consistent with our hypothesis and the evidence for other screening types. For example, living further from clinics or living in a neighborhood with lower density of screening facilities has been associated with poorer participation in mammography screening to detect breast cancer (Khan-Gates *et al.*, 2015). In contrast, the association between distance to polyclinic and block screening participation rate is susceptible to confounding and variation from accessibility to other facilities where we observed significant association in Models 1 and 3 only. Among the two primary care facilities considered, polyclinics were less accessible with median distance of 2.1 km compared to <1 km for subsidized private clinics, which suggests that when primary care facilities are within walking distance to one’s residence, they have higher potential to influence door-to-door screening participation.

Meanwhile, the finding on park was encouraging as it suggests higher awareness among those living further away from park leading to higher door-to-door screening participation. Interestingly, we found no association between access to healthy eatery and block screening participation rate. A possible reason for this observation could be the lack of purchasing power to buy food from these healthy eateries (Morris *et al.*, 2014) and the potential dependence on food bank (The Food Bank Singapore Ltd, 2015), hence resulting in the lack of association with health behavior such as participation in CVD risk screening.

Our study has limitations. First, the non-participants were unavailable and hence blocks were used as the unit of analysis instead of individuals. To estimate participation rate per 100 apartment-years, we assumed that the composition of residents were similar across each apartment over the three consecutive years. There is likely small changes in the composition of residents given the short period of three years as the Ethnic Integration Policy (EIP) imposes ethnicity quota to maintain good ethnic mix in public housing estates (Housing & Development Board Singapore, 2017). Second, the door-to-door screening in our study was performed by trained volunteers of the Singapore Heart Foundation in housing blocks resided by population with low SES. Hence, our findings may not be generalizable to other populations in the urban setting, as well as to other form of health screening such as those performed by healthcare professionals for chronic diseases in a health facility. Next, we estimated the distances to the nearest facilities via a straight line between the block and facility coordinates instead of the actual route participants would take. Straight-line distances have been shown to be reasonable proxies of actual walking distances (Bliss *et al.*, 2012), especially since the distances to facilities in this study (except polyclinics) were short. Similar to other studies, our study could only demonstrate associations but not causation. Our study also lacked data on utilization of these health-promoting facilities to verify the findings. Future studies may consider investigating the effect of both environmental determinants and individual behavior on health screening participation. Although our database had data on self-reported history of high blood pressure, high cholesterol, and diabetes, it lacked data on co-habitation and mental health problems. Hence, our analyses only adjusted for age, gender, and ethnicity, and we could not discount the possibility of confounding. Lastly, 21% of the participant count in HME (*n* = 2619) in our sample had to be excluded due to missing data and discrepancies in self-reported and calculated ages. We performed two sensitivity analyses that did not exclude participants with discrepancies between self-reported and calculated ages by using age calculated from date of birth and self-reported age which reduced the loss of participation count to 5% (*n* = 2488) and 11% (*n* = 2330) respectively. Sensitivity analyses yielded similar findings (see Appendices 1 and 2).

Nevertheless, our study illustrates how integrating additional data from publicly available information allowed us to assess whether access to health-promoting facilities could affect participation in CVD risk screening, and how the findings could inform future planning of door-to-door screenings in urban settings. Future studies should also be conducted in other urban settings to corroborate our findings.

## Conclusion

Among the four health-promoting facilities considered in this study, access to subsidized private clinic and park was associated with participation in door-to-door CVD risk screening in a region with high population density. Hence, this suggests that the physical environment may influence CVD risk screening behavior in low SES populations. These trends in screening behavior among populations with low SES could advise planning of future screening initiatives in urban settings for populations with low SES. Future studies could examine associations between access to and actual utilization of these health-promoting facilities.

**What is already known on this subject:**

- Populations with low socioeconomic status (SES) not only face barriers to cardiovascular disease (CVD) risk screening, but have also been shown to have higher incidence and mortality of CVDs.
- A wide range of individual factors have been shown to be associated with CVD risk screening participation, but little attention has been given to physical environment factors.
- Associations between physical environment and health behavior such as diet and physical activity have been well studied, but not with health screening behavior.

**What this study adds:**

- This is the first study to examine the associations between access to health-promoting facilities and health screening participation among populations with low SES by integrating data from different sources.
- This study demonstrated that these associations varied based on the type of health-promoting facilities and the regions the blocks were located in.
- Our analyses illustrate how access to health-promoting facilities could influence participation in CVD risk screening and how it could advise the planning of future door-to-door screening initiatives in an urban setting for low SES population.

## Supplementary material

For supplementary material accompanying this paper visit https://doi.org/10.1017/S1463423619000318. click here to view supplementary material
