# Supplementary material for: Association between access to health-promoting facilities and participation in cardiovascular disease (CVD) risk screening among populations with low socioeconomic status (SES) in Singapore
Source: Prim Health Care Res Dev. 2019 Jul 1;20:e98. doi: 10.1017/S1463423619000318 (PMC6609976; doi:10.1017/S1463423619000318)
Supplement: Supplementary file 1 [file S1463423619000318supp001.docx]

| **Appendix 1. Sensitivity analyses using self-reported age without considering discrepancy with calculated age (N = 2488)** | | | | | | | | | | |
| --- | --- | --- | --- | --- | --- | --- | --- | --- | --- | --- |
| **Homogeneous effect** | **Model 1** | | | | | **Model 2** | | | | |
|  | **IRR** | **(95% CI)** | | | **P-value** | **IRR** | **95% CI** | | | **P-value** |
| Distance to the nearest: |  |  |  |  |  |  |  |  |  |  |
| Subsidized private clinic (in 100m) | 0.91 | (0.82 | - | 1.00) | 0.061 | 1.01 | (0.93 | - | 1.09) | 0.778 |
| Park (in km) | 0.93 | (0.62 | - | 1.40) | 0.735 | 1.05 | (0.80 | - | 1.38) | 0.712 |
| Healthy eatery (in 100m) | 1.03 | (0.95 | - | 1.11) | 0.054 | 1.02 | (0.96 | - | 1.09) | 0.483 |
| Polyclinic (in km) | **0.94** | **(0.90** | **-** | **0.98)** | **0.003** | **0.97** | **(0.94** | **-** | **1.00)** | **0.026** |
| **Heterogeneous effect** | **Model 3** | | | | | **Model 4** | | | | |
|  | **IRR** | **(95% CI)** | | | **P-value** | **IRR** | **95% CI** | | | **P-value** |
| Distance to the nearest subsidized private clinic (by every 100m) |  |  |  |  |  |  |  |  |  |  |
| East region | **1.47** | **(1.10** | **-** | **1.97)** | **0.009** | 1.35 | (0.57 | **-** | 3.21) | 0.491 |
| Central/West regions | 0.97 | (0.83 | - | 1.14) | 0.726 | 0.99 | (0.84 | - | 1.16) | 0.876 |
| North/North-East regions | 1.00 | (0.94 | - | 1.07) | 0.895 | **0.87** | **(0.79** | **-** | **0.97)** | **0.012** |
| Distance to the nearest park (by every km) |  |  |  |  |  |  |  |  |  |  |
| East region | 1.42 | (0.67 | - | 3.03) | 0.364 | 1.16 | (0.29 | **-** | 4.64) | 0.83 |
| Central/West regions | 0.86 | (0.62 | - | 1.22) | 0.403 | 0.84 | (0.61 | - | 1.17) | 0.31 |
| North/North-East regions | **1.37** | **(1.06** | **-** | **1.76)** | **0.014** | **1.91** | **(1.15** | **-** | **3.18)** | **0.013** |
| Distance to the nearest healthy eatery (by every 100m) |  |  |  |  |  |  |  |  |  |  |
| East region | 1.04 | (0.83 | - | 1.31) | 0.741 | - | - | - | - | - |
| Central/West regions | 1.00 | (0.94 | - | 1.06) | 0.917 | - | - | - | - | - |
| North/North-East regions | 1.07 | (0.96 | - | 1.19) | 0.241 | - | - | - | - | - |
| Distance to the nearest polyclinic (by every km) |  |  |  |  |  |  |  |  |  |  |
| East region | 0.80 | (0.62 | - | 1.04) | 0.090 | - | - | - | - | - |
| Central/West regions | 1.01 | (0.96 | - | 1.05) | 0.747 | - | - | - | - | - |
| North/North-East regions | **0.93** | **(0.89** | **-** | **0.97)** | **0.002** | - | - | - | - | - |
| Model 1: Unadjusted analysis for each facility  Model 2: Adjusted for age, gender, ethnicity and region with homogeneous effect for each facility  Model 3: Adjusted for age, gender and ethnicity with heterogeneous effect across region for each facility  Model 4: Adjusted for age, ethnicity, gender and distance to the nearest polyclinic with heterogeneous effect across regions for private clinic and park  IRR: Incidence Rate Ratio | | | | | | | | | | |
| **Appendix 2. Sensitivity analyses using calculated age without considering discrepancy with self-reported age (N = 2330)** | | | | | | | | | | |
| **Homogeneous effect** | **Model 1** | | | | | **Model 2** | | | | |
|  | **IRR** | **(95% CI)** | | | **P-value** | **IRR** | **95% CI** | | | **P-value** |
| Distance to the nearest: |  |  |  |  |  |  |  |  |  |  |
| Subsidized private clinic (in 100m) | 0.91 | 0.83 | - | 1.01 | 0.071 | 1.02 | (0.95 | - | 1.10) | 0.629 |
| Park (in km) | 0.95 | 0.65 | - | 1.40 | 0.796 | 1.08 | (0.84 | - | 1.39) | 0.555 |
| Healthy eatery (in 100m) | 1.02 | 0.95 | - | 1.11 | 0.570 | 1.02 | (0.96 | - | 1.08) | 0.586 |
| Polyclinic (in km) | **0.93** | **0.89** | **-** | **0.97** | **< 0.001** | **0.96** | **(0.93** | **-** | **0.99)** | **0.015** |
| **Heterogeneous effect** | **Model 3** | | | | | **Model 4** | | | | |
|  | **IRR** | **(95% CI)** | | | **P-value** | **IRR** | **95% CI** | | | **P-value** |
| Distance to the nearest subsidized private clinic (by every 100m) |  |  |  |  |  |  |  |  |  |  |
| East region | **1.40** | **(1.06** | **-** | **1.86)** | **0.018** | 1.32 | (0.59 | **-** | 2.96) | 0.498 |
| Central/West regions | 0.98 | (0.84 | - | 1.15) | 0.810 | 1.00 | (0.85 | - | 1.16) | 0.955 |
| North/North-East regions | 1.02 | (0.95 | - | 1.09) | 0.611 | **0.86** | **(0.79** | **-** | **0.94)** | **0.001** |
| Distance to the nearest park (by every km) |  |  |  |  |  |  |  |  |  |  |
| East region | 1.32 | (0.65 | - | 2.71) | 0.445 | 1.09 | (0.30 | **-** | 3.96) | 0.892 |
| Central/West regions | 0.90 | (0.65 | - | 1.25) | 0.542 | 0.88 | (0.64 | - | 1.20) | 0.414 |
| North/North-East regions | **1.51** | **(1.19** | **-** | **1.93)** | **< 0.001** | **2.16** | **(1.40** | **-** | **3.35)** | **0.001** |
| Distance to the nearest healthy eatery (by every 100m) |  |  |  |  |  |  |  |  |  |  |
| East region | 0.99 | (0.79 | - | 1.24) | 0.928 | - | - | - | - | - |
| Central/West regions | 1.00 | (0.94 | - | 1.06) | 0.980 | - | - | - | - | - |
| North/North-East regions | 1.07 | (0.96 | - | 1.20) | 0.203 | - | - | - | - | - |
| Distance to the nearest polyclinic (by every km) |  |  |  |  |  |  |  |  |  |  |
| East region | 0.92 | (0.74 | - | 1.15) | 0.479 | - | - | - | - | - |
| Central/West regions | 1.00 | (0.96 | - | 1.04) | 0.943 | - | - | - | - | - |
| North/North-East regions | **0.92** | **(0.87** | **-** | **0.96)** | **< 0.001** | - | - | - | - | - |
| Model 1: Unadjusted analysis for each facility  Model 2: Adjusted for age, gender, ethnicity and region with homogeneous effect for each facility  Model 3: Adjusted for age, gender and ethnicity with heterogeneous effect across region for each facility  Model 4: Adjusted for age, ethnicity, gender and distance to the nearest polyclinic with heterogeneous effect across regions for private clinic and park  IRR: Incidence Rate Ratio | | | | | | | | | | |
